# New products

**DOI:** 10.1155/S1463924692000075

**Published:** 1992


Journal of Automatic Chemistry, Vol. 14, No. 1 (January–February 1992), pp. 29–34

### New products

### LEMAS

Link Analytical's LEMAS is for use with Scanning Electron Microscopes (SEMs). Besides the substantial automation advantages of increased sample throughput and 24-hour operation, LEMAS offers the benefit of an extremely quick and simple way to both set-up and manually operate the stage and WD spectrometers. The operator uses a small control console with a combined electroluminescant display and touch-pad plus two joysticks. A single press of the touch-pad calls the appropriate page to set-up both stage and spectrometer positions. A joystick controls all stage movements; a separate joystick selects spectrometer wavelength. LEMAS has a modular design based around a highly efficient distributed processing architecture. So it can be upgraded in the field by simply slotting in new boards; extra spectrometer channels or stage axes can be added as the demand grows.

The system uses dedicated microprocessors to control each of the microscope's motor drives, and additional microprocessors are used for the spectrometer's counting electronics. This speeds up the automation process, for example by allowing analysis from one spectrometer while another one is being repositioned. It guarantees both smoothness and the sensitive control necessary when locating a specimen to within a micron. The system also guarantees fast X-ray throughput, rapid wavelength selection and extremely high reproducibility.

*Details from Janet Wood, Link Analytical Limited, Halifax Road, High Wycombe, Buckinghamshire HP2 3SE, UK.*

### Peak detector

The PeakTrak Peak Detector/Autozero module is a high performance peak monitor for fast, accurate fraction collection or process monitoring. Sampling a detector signal at 200 Hz in real time, PeakTrak senses leading peak edges earlier than other peak detectors, with user-programmable thresholds. It can be utilized to switch a collector, alarm, or other device in response to a peak's appearance, and can be controlled by an external computer or integrator to monitor selected time windows only. In addition, PeakTrak provides automatic autozero capability for any existing detector. Axxiom is offering qualified institutions a free 30-day PeakTrak trial upon request.

*Details from Axxiom Chromatography, Inc., 11988 Challenger Court, Moorpark, California 93021-7122, USA. Tel.: 805 523 8888.*

### Trace CFCs

An Application Note from Hewlett-Packard (available from the company free of charge) describes the analysis of air samples containing parts-per-billion traces of chlorofluorocarbons (CFCs) using a GC/MS system based on the HP MS Engine, a state-of-the-art research mass spectrometer. The analytical method is timely in view of increasing concern about the ozone layer.

*Details from Verena Haller, Hewlett-Packard SA, 150 Route du Nant-d'Avril, CH-1217 Meyrin (GE) 2, Switzerland.*

### p$K_a$ and logP measurement

Sirus Analytical Instruments has launched an instrument for measurement of p$K_a$ and logP (octanol/water partition coefficient), two physical chemistry parameters of importance in QSAR studies. Called the PCA 101, the instrument uses a potentiometric technique to collect experimental data, from which p$K_a$ and LogP are calculated by a computer-based mathematical procedure. p$K_a$s from 1·5 to 12·0 can be determined

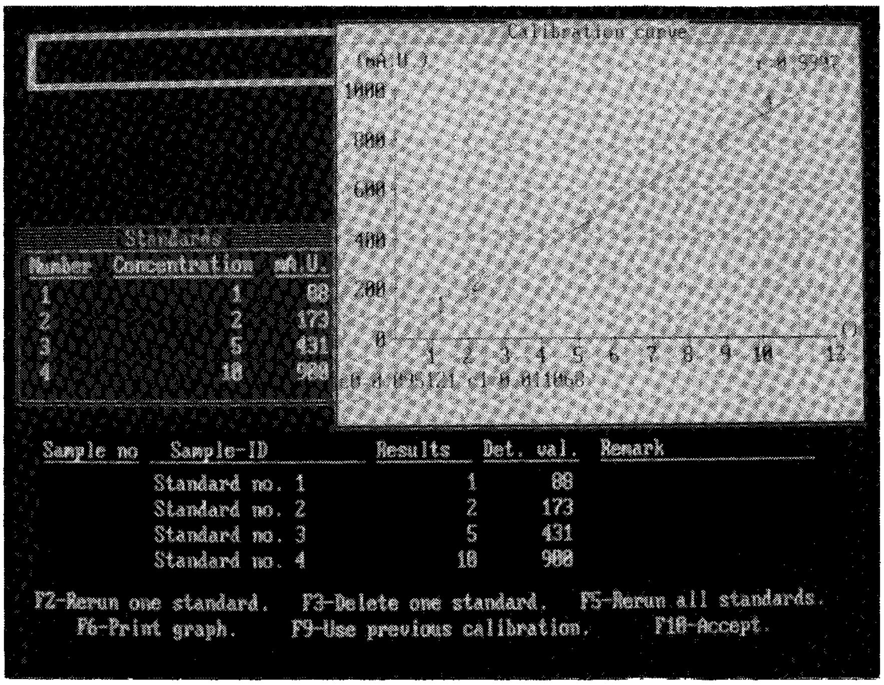

*SuperFlow EX is the third generation software for flow injection analysis. The software makes it possible to integrate the Tecator FIAstar 5010 system with virtually any type of flow-through detector. It is especially suitable for UV-Vis, fluorescense and luminescense detectors. Detectors with an output signal of up to 10 V are easily integrated into the FIA system. Maximum resolution from the detector is ensured by an automatic range-switching function in the software. This gives maximum sensitivity, combined with a range eight times better than conventional systems. More information from Tecator AB, Box 70, S 263 21 Höganäs, Sweden.*

accurately (values which are well outside the range of simpler approaches); logP values up to 5·0 are the highest that have been measured to date using the instrument, but preliminary results suggest that higher values can be measureable, provided samples have at least one ionizable functional group.

The instrument is fully automated; the user weighs a sample of pure material (typically 1 to 50 mg) into a vial, and loads, it; the instrument then takes over, adding water and solvent/s, and titrating with acid and base. Typical analysis time is 25 min to determine $pK_a$, and 45 min for $pK_a$ and logP. Work is in progress to develop a faster version of the instrument.

Results are calculated using a built-in program, in which variables like sample weight, molecular weight, quantities of acid and base used. pH electrode response and calibration data are analysed, by non-linear regression to establish and refine $pK_a$ and logP values. The program is powerful, and has been enhanced through the study of challenging samples such as compounds with several $pK_a$s, mixtures with overlapping $pK_a$s, and very insoluble materials. Using a calculation based on Bjerrum difference plots, $pK_a$s can be determined for compounds which decompose over a particular region of the pH scale, even if the $pK_a$ lies in the unstable region. The technique of measuring logP, while giving results comparable to the shake-flask method, is especially useful for zwitterions and problems associated with ion-pair formation, and for compounds which suffer from micro-emulsion problems.

*$pK_a$ and logP analytical service*

The Sirius Applications Laboratory is running an analytical service for $pK_a$ and/or logP determination. To use this service, please contact Sirius for full information and an order form/questionnaire. The service aims to receive samples by mail, and to send results within a few days of receipt.

*Details from Sirius Analytical Instruments Ltd, Manor House, Lewes Road, Forest Row, East Sussex RH18 5AF, UK. Tel.: 0342 824036.*

### Monitoring hazardous substances

The 1991 update of the database which provides employers with information on how to measure airborne concentrations of hazardous substances in the workplace was published in August by the Health and Safety Executive (HSE). Employers are required in certain circumstances to carry out such measurements under the Control of Substances Hazardous to Health Regulations 1988 (COSHH).

The revised database – 'EH40: Workplace Air and Biological Monitoring Database 1991' – has been developed jointly by HSE's Occupational Medicine and Hygiene Laboratory and Sheffield University's Department of Information Studies.

The reference to 'EH40' relates to the HSE Guidance Note EH40, a booklet which gives details of the occupational exposure limits for hazardous substances. The information on the database enables users to decide how best to monitor the presence of such substances on their premises using the resources available to them – such as equipment or laboratory facilities.

The program was designed with ease of use in mind, and it runs under DOS v.2 to DOS v.3.3 on fully IBM-compatible PCs having a minium of 640 Kb of memory and a hard disc with 2.5 Mb of free space, together with a $5\frac{1}{4}$ in or $3\frac{1}{2}$ in floppy disc-drive to load the program.

*'EH40 Workplace Air and Biological Monitoring Database 1991' is available from HMSO, price £200 plus VAT. Subsequent annual updates will be available at a discounted cost to continuous subscribers. Database pack with $3\frac{1}{2}$ in discs: ISBN 0-11-885606-5. Database pack with $5\frac{1}{4}$ in discs: ISBN 0-11-885607-3.*

### Automated process analysers

AEA Technology at Dounreay, has set up a Process Instrument Development Department whose aims are to introduce on-line and near-line sensors and analytical instrumentation into the Dounreay reprocessing/fuel production plants and test rigs. The establishment recognizes the benefits of process instrumentation and cites the main advantages as the reduction of the number of samples which have to be taken and analysed manually in the laboratory. Also, automated process instrumentation gives the plant operators the benefit of very frequent (or even continuous) analytical results on a near real-time basis.

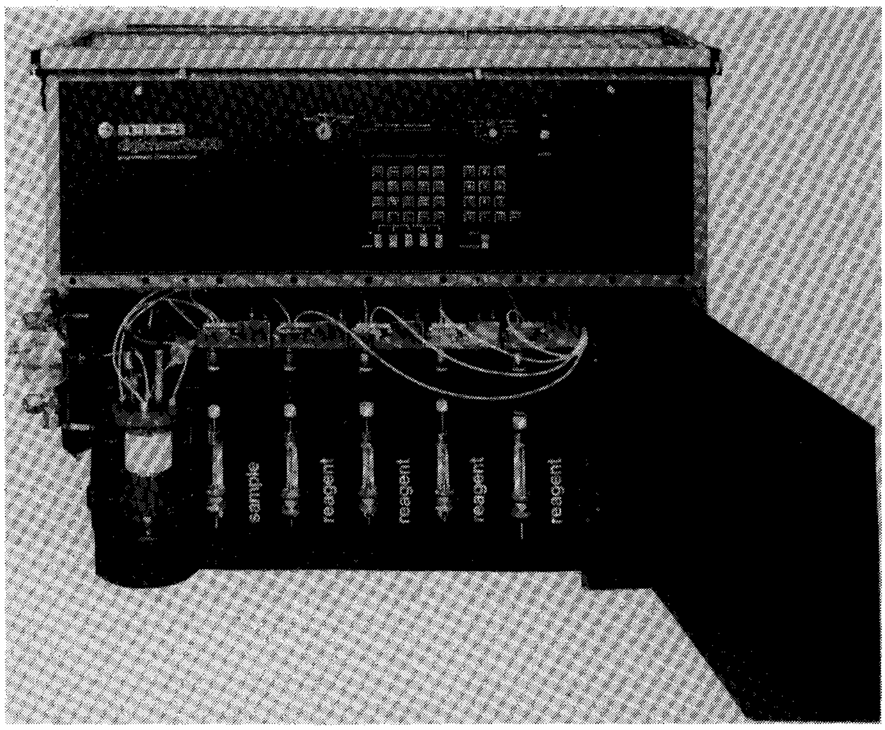

*Ionics' Digichem 3000 used at Dounreay for analyses of trace uranium, hydrazine and mixed nitric/sulphuric acids.*

These measurements can then be used for timely plant-control purposes, hence leading to increased plant efficiency.

Work includes the design, development, calibration, testing and implementation of process sensor systems for installation on plant streams. Installed process sensors include those for making physical measurements (such as density, flow rates, temperature, conductivity) and also instruments for performing chemical determinations. Automatic wet chemical analysers include the Ionics Digichem 3000 process autoanalyser used in analyses for trace uranium, hydrazine and mixed nitric/sulphuric acids.

The Digichem 3000 is a rugged, plant based autoanalyser capable of performing a wide range of colorimetric titrimetric and ion-selective electrode based analyses.

Samples and reagents are aspirated by stepper motor driven syringes. The syringes then operate as accurate burettes and dispense samples and reagents into a slowly rotating vessel. The slow rotation, combined with the effect of the dispenser tips and sensors dipping into the solution ensures efficient mixing and rapid reaction.

The solution is then interrogated by the sensor probes (pH or REDOX electrodes for titrations, fibre-optic immersion probe for the colorimetry, or ion-selective electrodes for direct concentration measurements). Sensor signals then pass via the sensor evaluator to the computer which calculates and prints out the results.

Finally, the rotating reaction vessel spins rapidly to empty its contents by centrifugal force into an outer drain housing. The cup is then washed, emptied and spun dry in readiness for the next sample.

All the above operations are performed automatically under the control of the instrument's dedicated computer and the instrument can be left unattended, sampling at intervals of as little as 10 minutes, for periods of at least a week.

*For further details of the Digichem 3000 contact Ionics UK Ltd, Carrington Business Park, Carrington, Urmston, Manchester M31 4DD, UK.*

### Bar-coding on the bus

First in a significant new series of add-on products to increase the capability of Apple Macintosh computers equipped with Apple DeskTop Bus (ADB), the BC-91 Bar Code Reader provides a simple cost-effective means of implementing automated database applications. Compatible

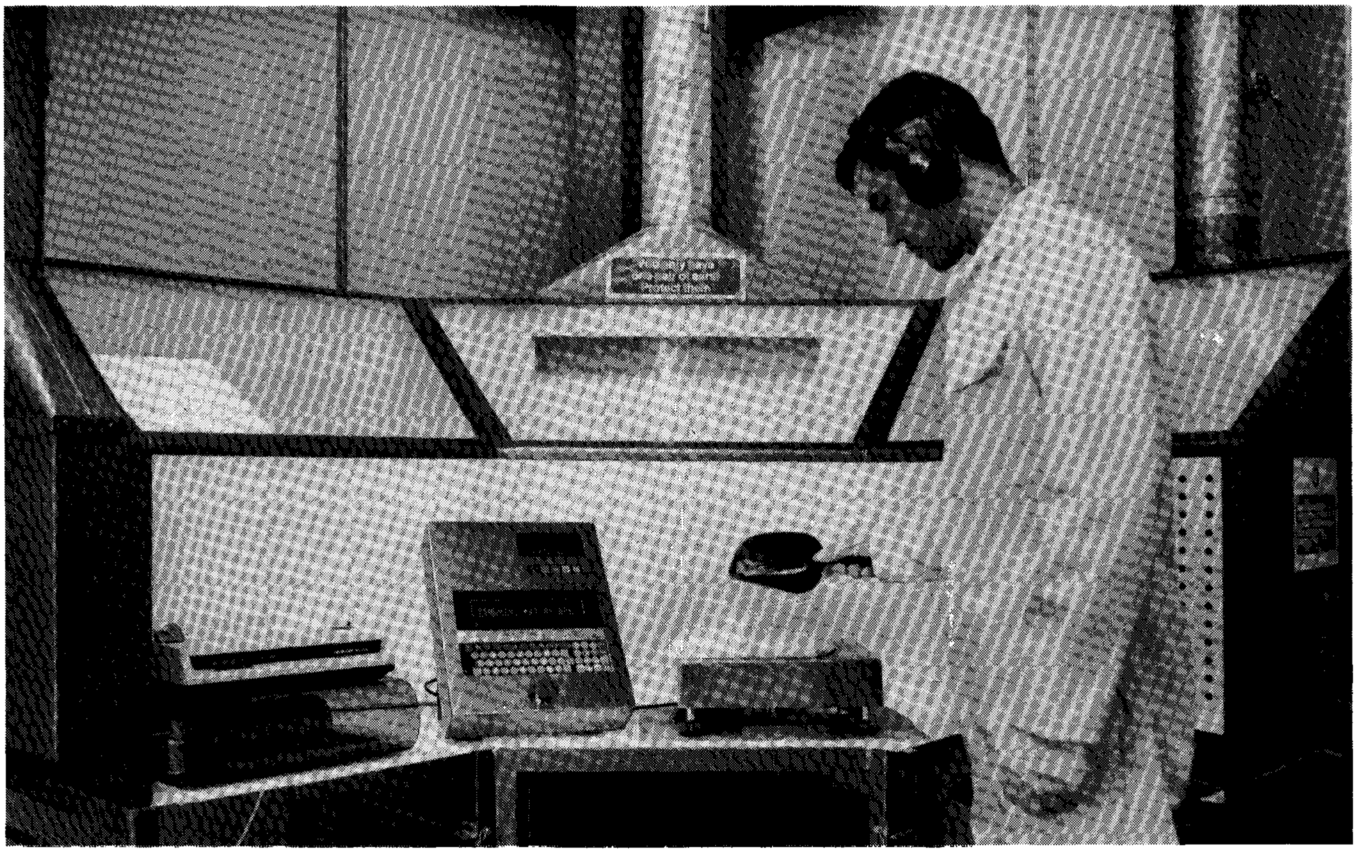

*Mettler-Toledo's new recipe formulation equipment forms an important ingredient in Wells Plastics' bid for BS 5750 quality approval. Comprising a stainless-steel bench scale, programmable indicator and report printer, the package runs on standard software able to control and monitor the weighing of, typically, 100 raw materials and recipes in a virtually unlimited range of process industries.*

*Wells Plastics has recently installed two systems to formulate polymer compounds for additive masterbatches being produced in pellet form on the company's extrusion lines in Stones, Staffordshire, UK.*

*Mettler-Toledo has also supplied identical systems to several major food processors in recent months. Details from Mettler-Toledo, 64 Boston Road, Beaumont Leys, Leicestershire LE4 1AW, UK.*

with all existing Macintosh software, it allows bar codes to be used for inventory control, point-of-sale, database management, accounting and invoicing and many other applications, without the need for the custom programming.

*Enquiries to Paul Bragg, Adept Scientific Micro Systems Ltd, 6 Business Centre West, Avenue One, Letchworth, Hertfordshire SG6 2HB, UK. Tel.: 0462 480055.*

### Engine oil monitoring

Monitoring the Total Acid Number (TAN) and Total Base Number (TBN) in marine and power plant engine oils provides early warning of additive break-up – the oil can be changed in time and the risk of engine damage is avoided. Radiometer's TitraLab Automated Titration Laboratory can be fitted with a TAN/TBN Method Module for performing titration of engine and other oils according to ASTM D 664. Titration results can be taken from both inflection points and the fixed potentials found. So the Strong Acid Number (SAN) and TAN or Strong Base Number (SBN) and TBN can be determined in only one titration. Results are then automatically calculated in mg KOH/g.

The Tributette Station of TitraLab holds three different reagents suitable for the solvent and the two different titrants used for TAN/TBN determinations according to ASTM D 664. TitraLab also automatically adds solvent to the sample before the titration.

Up to 60 methods can be stored on TitraLab, with the electrodes and burettes to be used preprogrammed. The basic system can also be linked up with a sample charger, balance, and robot or external PC.

*For more information contact Ed Lemon at Radiometer Ltd, The Manor, Manor Royal, Crawley, West Sussex RH10 2PY, UK. Tel.: 0293 517599.*

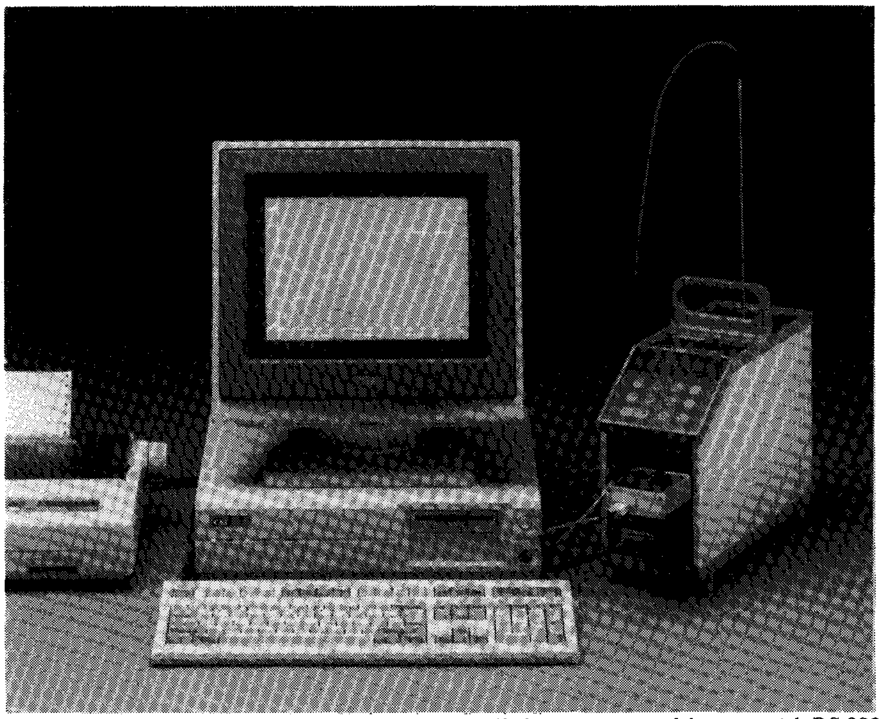

*An updated version of Jofra's microprocessor controlled temperature calibrators with RS 232 C interface, including full hardware and software packages is available. The user can configure and save them for later documentation and/or comparision, for proof of quality assurance. A line of input modules for T/C, EJKRST, PT100, PT1000 and voltage signals are available, enabling the user to run a complete test directly controlled from the PC keyboard. The RS 232 C interface is available for all JOFRA temperature calibrators producing temperatures between −50 °C to +650 °C. The calibrators are supplied with an internationally traceable certificate.*

*For further information contact Ametek Denmark A/S, Gammelgårdsvej 87, DK 3520 Farum, Denmark.*

### UV/Vis Spectrophotometers

Unicam has produced two brochures describing its UV/Vis spectrophotometers. The first brochure illustrates the PU8730 Professional-UV series. Four models are described with fixed or variable bandwidth with a high-resolution colour or monochrome VDU, together with a mouse-driven graphic user interface. Sample handling accessories and method set-up and data processing screens are also pictured.

The second brochure is devoted to Unicam's PU8710 Personal-UV instruments. This series comprises the PU8710, a fixed bandwidth unit, and the PU8715, a more sophisticated variable bandwidth model for higher resolution work. Both spectrophotometers are illustrated working with Unicam's Falcon software on an IBM compatible PC. Disk data and method storage examples are shown together with accessories and hard copy options.

*Further information from Paul Carter, Unicam Ltd, York Street, Cambridge CB1 2PX, UK. Tel.: 0223 358866.*

### IR polymer libraries

Heyden & Son have announced the availability of an eight page brochure describing in detail a collection of infra-red polymer search libraries. The 17 libraries cover all the various polymer chemistry fields. All spectra have been selected and reviewed by experts in these fields. Details of the chemical classifications in each library are also given in the brochure.

*For further details contact Jeff Seymour, Heyden & Son Ltd, Spectrum House, Hillview Gardens, London NW4 2JQ. Tel.: 081 203 5171.*

### Trace 0/2 Analyser

The Model 3160 is Teledyne's latest addition to its line of trace oxygen (0/2) analysers. The Model 3160 incorporates a microprocesser that provides versatile control of user-selectable analogue or digital outputs on high or low alarms. The 19-in rack-mounted Model 3160 features *AutoRanging* over four ranges as low as 0–1 ppm. The 3160 is easy to use, with self-explantatory operation and an easy-access help menu; it offers full-scale accuracy (+2%) and fast response (90% of 1000–10 000 ppm in less than 10 s).

The 3160 will be suited to online monitoring of trace oxygen in nitrogen, argon, hydrocarbons, hydrogen, and many other pure gases and gas mixtures. Gas monitoring with the 3160 is important for process control, quality assurance and process protection in a wide variety of industrial applications.

*Details from Teledyne Analytical Instruments, Harlequin Centre, Southall Lane, Southall, Middlesex UB1 5NH. Tel.: 081 571 9596.*

### Advanced gas detection technology

British Gas is co-funding a £300 000 project with Morgan Crucible aimed at developing highly sensitive ceramic sensors for detecting and monitoring the presence of a wide range of gases in industrial and domestic applications.

The three-year joint collaboration is being carried out by British Gas at its Watson House Research Station with two Morgan Crucible subsidiaries, Morganite Electronic Instruments Ltd and Morgan Materials Technology Ltd., as well as the University of Wales, Swansea, under the Department of Trade and Industry 'LINK' Molecular Sensors Programme. The project is partly funded by the Department of Trade and Industry to encourage collaboration between universities and industry in work of this kind. Under the scheme, support for the project is also provided for the University of Wales by the Science and Engineering Research Council.

There are many established techniques for monitoring gases in industrial processes or in the laboratory, for use in applications such as safety monitoring or alarms, routine analysis or process control. However, instrumentation is often sophisticated and costly as in the measurement, for example, of carbon monoxide, using infra-red absorption or chromatographic techniques or short-lived sensors such as electrochemical cells. In principle, extremely simple low-cost sensors offering long life can be used in a large proportion of applications, but these suffer from poor selectivity to certain gases and are unreliable. They also have a high power consumption restricting their use in portable battery powered equipment.

British Gas has been actively involved in investigating a number of developments in the search for a reliable gas detector, through work at its research stations and support given to the University of Wales. Existing research on a number of these sensors for detecting gases such as methane, carbon monoxide and hydrogen has now reached a significant stage of development. A better understanding has been achieved in the relationship between sensors and substrate fabrication techniques and this has enabled the project to be carried forward a further stage.

The joint collaboration will develop pre-production quality sensors involving polycrystalline semi-conductor technologies for assessment in detecting and monitoring equipment.

*For further information contact British Gas, Rivermill House, 152 Grosvenor Road, London SW1V 3JL. Tel.: 071 821 1444.*

### Consistent shot volume

A traditional problem in automatic dispensing systems is that as the dispensing barrel becomes exhausted of material, so there is a reduction of the pressure in the barrel itself, and, consequently, a fall in dispensing precision and shot volume. A dispensing system from Hakuto International overcomes this problem by automatically compensating for this pressure decrease. This feature makes it unique among both automatic and manual dispensing systems currently available.

The Accura I is based on a microcomputer with non-volatile storage of user-defined shot time settings for up to four channels. It may be programmed to deliver a specified number of shots via any one or a combination of channels, and generates an alarm signal when each shot cycle is completed. The shot time range may be set between 0·001 and 9·999 s, or, as selected by an internal DIP switch, 0·01 and 99·99 s. Three alternative operating modes are available: 'timed' or 'steady', providing either metered shots or a continuous flow; and 'remote', under control from an external device or system.

Because the Accura I is designed for use with fluids and materials of wide-ranging viscosities and may be fitted with all commonly used dispensing nozzles, it is essential that the shot volume is maintained constant regardless of the dispensing barrel content, the material being used, and the nozzle size. Accordingly, the Accura I incorporates a unique controller which provides continuous pressure compensation, such that the pressure within the barrel, normally between 0·09 and 4·8 bar, is automatically increased as the material content decreases, until the barrel becomes exhausted.

The Accura I operates from a standard a.c. mains power supply at 50/60 Hz, and, although the need for an oil-free dry air supply of between 5·4 and 5·8 bar is specified, the system also contains a novel combination filter to ensure that the air supply to the dispensing barrel is of extremely high purity. It is provided with a foot switch for local control, and can be incorporated into an automated production line.

*Details from Hakuto International, Eleanor House, 33–35 Eleanor Cross Road, Waltham Cross, Hertfordshire EN8 7LF, UK. Tel.: 0992 769090.*

### Measurement of TOC, POC, TC

Ionics UK Ltd has announced a laboratory water analyser capable of Total Organic Carbon (TOC), Purgeable Organic Carbon (POC) and Total Carbon (TC) measurement from 10 ppb to 2000 ppm. The model 1505 is designed for laboratory applications in a variety of industries including municipal and industrial waste treatment, industrial process plants, chemicals, petroleum, pulp and paper, food processing and colleges and universities.

The new analyser uses a high temperature reaction system for the combustion of all organic species at 900 °C. An automatic sample injection system, featuring all-ceramic construction, introduces samples to the system. In each mode of operation, a sample is aspirated from a sample tube and injected into the high temperature reaction chamber. The sample is oxidized and all carbonaceous material is converted to carbon dioxide. A high-sensitivity infra-red analyser measures the concentration of carbon dioxide and displays the ppm or ppb of carbon present on a direct-reading digital display. The same signal can also be sent to a remote recorder or transmitted to a computer or data logger.

When performing Total Organic Carbon or Total Carbon analyses, a high-sensitivity, non-dispersive infra-red detector is used to specifically measure carbon dioxide. TOC measurements are made by adding a few drops of acid to the sample to be sparged. POC measurements are made by adding base to the sample to be sparged. Sparging is automatic with the instrument in the TOC or POC operation mode.

*For more information contact Ionics UK Ltd, Carrington Business Park, Carrington, Urmston, Manchester M31 4DD, UK.*

### Three detectors in one

A high performance liquid chromatograph system combining three of the most popular detection modes—UV, fluorescence and conductivity—into a single, compact unit, is now available from Bacharach, Inc. Called the Coleman 3D HPLC, it is being promoted to laboratories for QC, screening and teaching. The unit is supplied with pump, injector, cartridge column, recorder and trifunctional detector. Minimal set up is required and periodic maintenance can be done in the laboratory; the price is under $7000.

*Details from Bacharach, Inc. 625 Alpha Drive, Pittsburgh, Pennsylvania 15238, USA. Tel.: 412 963 2000.*

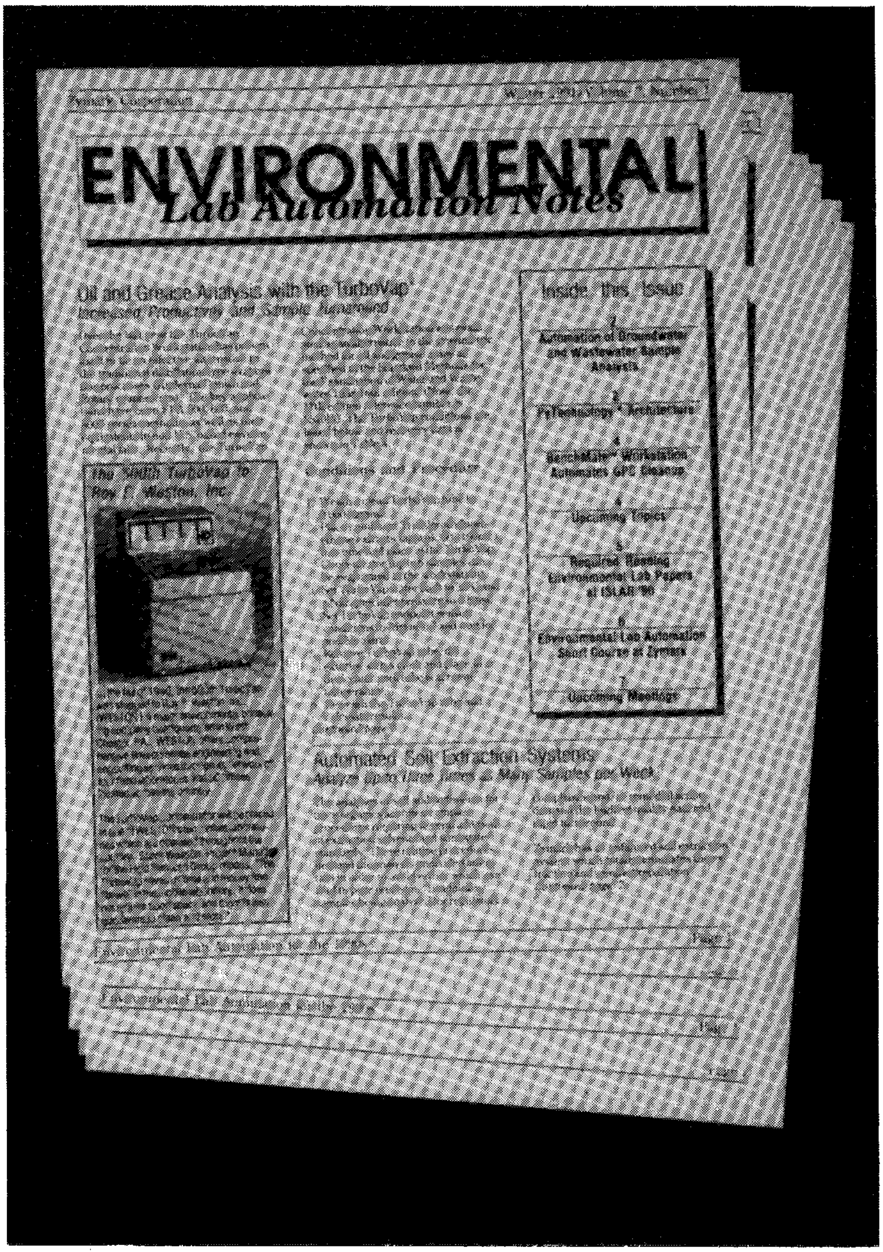

*Zymark is now publishing a newsletter: 'Environmental Lab Automation Notes', to assist scientists and managers in the environmental industry in keeping up to date with recent developments in laboratory automation. The newsletter reviews recent regulatory developments in the environmental labs, many of which strongly impact its productivity and certification. The newsletter includes updates on new product developments from Zymark and also includes excerpts from recent publications on laboratory automation topics by other scientists and managers in the environmental industry. Winter 1991's newsletter includes: GPC cleanup for environmental extracts; Pesticide analysis using large volume solid phase extraction; and solvent reclamation using the TurboVap 500. Details from Sharon Correia, Zymark Corporation, Hopkinton, Massachusetts 01748, USA.*

